# Cycloid psychoses and autoimmunity: A case report of a patient with motility psychosis and Hashimoto’s thyroiditis

**DOI:** 10.1192/j.eurpsy.2022.1807

**Published:** 2022-09-01

**Authors:** M. Lages Abrantes, J. Borja Santos

**Affiliations:** Hospital Prof. Doutor Fernando Fonseca, Psychiatry And Mental Health Department, Amadora, Portugal

**Keywords:** cycloid psychosis, Hashimotto thyroiditis, autoimmunity

## Abstract

**Introduction:**

Psychotic episodes characterized by sudden onset of polymorphous psychotic symptoms and fast resolution have been a subject of interest of many psychiatrists throughout the History. Controversies about the diagnostic criteria and nomenclature of cycloid psychoses persist nowadays, what has hampered its study. In last years, several reports associating this disease with autoimmune pathologies have been published, revealing a possible association between them.

**Objectives:**

To contribute to the knowledge of cycloid psychoses, reporting a case of motility psychoses and exploring its possible association with autoimmune diseases.

**Methods:**

Case report and literature review.

**Results:**

A 48-years-old woman presents a history of eleven admissions at the Psychiatry nursery in the last nineteen years, due to psychotic episodes. Usually, these episodes follow a default in psychopharmacological therapeutic, and are characterized by rapid onset of psychomotor agitation, with prominent nonpurpuseful exuberant movements, incoherent speech, mood oscillations and polythematic delusion. Between these decompensations, she recover her normal functioning, being medicated with lithium and an antipsychotic. During one of her admissions, at 2015, she developed fever and a stuporous state. The magnetic resonance and lumbar puncture were normal, the electroencephalogram revealed generalized lentification. Autoimmunity investigation evidenced positive antithyroid antibodies (with normal thyroid function) and the echography validated the diagnosis of Hashimoto’s thyroiditis.

**Conclusions:**

This case report reveals a possible relationship between cycloid psychoses and Hashimoto’s thyroiditis. We need to share more knowledge to understand if it represents a comorbidity or a pathogenic process with the same etiology, what will influence the treatment of these patients.

**Disclosure:**

No significant relationships.

